# sPDGFRβ and neuroinflammation are associated with AD biomarkers and differ by race: The ASCEND Study

**DOI:** 10.1002/alz.13457

**Published:** 2023-11-06

**Authors:** Brittany Butts, Hanfeng Huang, William T. Hu, Patrick Gavin Kehoe, James Scott Miners, Danielle D. Verble, Henrik Zetterberg, Liping Zhao, Lynn Marie Trotti, Karima Benameur, Laura M. Scorr, Whitney Wharton

**Affiliations:** ^1^ Emory University Nell Hodgson Woodruff School of Nursing Atlanta Georgia USA; ^2^ Georgetown University, School of Medicine Washington District of Columbia USA; ^3^ Rutgers University Institute for Health, Health Care Policy, and Aging Research New Brunswick New Jersey USA; ^4^ University of Bristol Dementia Research Group Bristol UK; ^5^ Department of Psychiatry and Neurochemistry, Institute of Neuroscience and Physiology, the Sahlgrenska Academy at the University of Gothenburg, Mölndal, Sweden; Clinical Neurochemistry Laboratory, Sahlgrenska University Hospital, Mölndal, Sweden; Department of Neurodegenerative Disease, UCL Institute of Neurology, Queen Square, London, UK; UK Dementia Research Institute at UCL, London, UK; Hong Kong Center for Neurodegenerative Diseases, Clear Water Bay Hong Kong China; ^6^ Emory University Rollins School of Public Health Atlanta Georgia USA; ^7^ Emory University School of Medicine Atlanta Georgia USA

**Keywords:** Alzheimer's disease, parental history, prevention, tau, vascular risk

## Abstract

**INTRODUCTION:**

There remains an urgent need to identify preclinical pathophysiological mechanisms of Alzheimer's disease (AD) development in high‐risk, racially diverse populations. We explored the relationship between cerebrospinal fluid (CSF) markers of vascular injury and neuroinflammation with AD biomarkers in middle‐aged Black/African American (B/AA) and non‐Hispanic White (NHW) participants.

**METHODS:**

Adults (45–65 years) with a parental history of AD were enrolled (*n* = 82). CSF and blood biomarkers were collected at baseline and year 2.

**RESULTS:**

CSF total tau (t‐tau), phosphorylated tau (p‐tau), and amyloid beta (Aβ)40 were elevated at year 2 compared to baseline. CSF soluble platelet‐derived growth factor receptor β (sPDGFRβ) levels, a marker of pericyte injury, correlated positively with t‐tau, p‐tau, Aβ40 markers of vascular injury, and cytokines at baseline and year 2. CSF sPDGFRβ and tau were significantly lower in B/AA than NHW.

**DISCUSSION:**

Vascular dysfunction and neuroinflammation may precede cognitive decline and disease pathology in the very early preclinical stages of AD, and there are race‐related differences in these relationships.

**Highlights:**

- Cerebrospinal fluid (CSF) Alzheimer's disease (AD) biomarkers changed over 2 years in high‐risk middle‐aged adults.
- Markers of vascular dysfunction were associated with the CSF biomarkers amyloid beta and tau.
- AD biomarkers were lower in Black compared to non‐Hispanic White individuals.
- Markers of vascular dysfunction were lower among Black individuals.

## 1 INTRODUCTION

The increasing prevalence of Alzheimer's disease (AD) remains unaddressed due to the absence of effective disease‐modifying therapies, and there remains a significant and urgent need to identify preclinical pathophysiological mechanisms responsible for AD development in high‐risk, racially diverse populations. As pathophysiological mechanisms leading to the development of AD begin many years before clinical manifestations,^1^ there is a need to study biomarkers that reflect underlying pathological processes that precede the emergence of clinical symptoms in AD.^1^, ^2^ Accumulation of these biomarkers, defined by amyloid beta (Aβ) deposition, formation of fibrillar tau, and neurodegeneration (AT[N]), is a progressive and continuous process that begins years before the onset of clinical symptoms, often during midlife.^2^

Multifactorial putative mechanisms driving the neuropathology of AD, originally described by the amyloid cascade^3^ and cholinergic^4^ hypotheses, have led to the development of emerging complementary theories, including those that implicate vascular dysfunction,^5^ angiotensin II,^6^ and inflammation.^7^ Neurovascular dysfunction, including reduced cerebral blood flow and blood–brain barrier (BBB) breakdown, occur in early stages of AD.^8^, ^9^ Markers of endothelial injury are elevated in cerebrospinal fluid (CSF), serum, and pathological studies in AD.^10^, ^11^, ^12^ Renin–angiotensin system (RAS) dysregulation is implicated in AD pathology.^6^, ^13^ Increased angiotensin‐converting enzyme (ACE1) activity and decreased ACE2 activity have been reported in CSF and *post mortem* brains in AD and are associated with AD pathology.^11^, ^14^ Increased ACE1, proposed to play a role in Aβ degradation, is associated with higher levels of angiotensin II, which has multifactorial deleterious effects on several pathways associated with AD, including hypertension, vascular dysfunction, alterations in BBB function, and inflammation.^14^, ^15^ Angiotensin II–induced cytokine release is a likely contributor to AD pathology through amplification of neuroinflammation and activation of microglia.^7^, ^16^, ^17^

Pericytes appear to be crucial in maintaining vascular integrity and regulating cerebral blood flow and BBB permeability;^18^, ^19^ endothelial cell injury and damage to pericytes play a key role in AD pathology.^20^, ^21^ Pericytes and endothelial‐expressed matrix metalloproteinases (MMPs) and tissue inhibitor of metalloproteinases (TIMPs) are involved in vascular homeostasis and dysregulated in AD.^22^, ^23^ In AD *post mortem* brain tissue, there is significant pericyte loss and a reduction in platelet‐derived growth factor receptor β (PDGFRβ) levels.^24^, ^25^ Pericytes in culture shed soluble PDGFRβ (sPDGFRβ) under hypoxic conditions or exposure to Aβ peptides.^26^ Elevated CSF levels of sPDGFRβ have been associated with BBB breakdown within the hippocampus in mildly cognitively impaired participants^15^ and were reported to predict cognitive decline in early stages of AD independently of changes in Aβ and tau.^27^ We have previously shown that CSF sPDGFRβ levels were elevated from clinical AD cases and correlated with CSF albumin, a marker of BBB breakdown, and levels of CSF total tau (t‐tau) and phosphorylated tau (p‐tau).^28^

Black/African American adults (B/AAs) are 64% more likely to develop AD than non‐Hispanic White adults (NHWs).^29^ The higher incidence and prevalence of AD among B/AAs are often attributed to sociocultural issues and biological and genetic factors related to higher cardiovascular risk, such as diabetes and hypertension. More work is needed to examine race‐associated differences in AD biomarkers as these differences may further contribute to health disparities in AD diagnosis and potential success of preventive interventions.

Recent studies in B/AA and NHW adults found that CSF levels of t‐tau and p‐tau are lower in cognitively impaired B/AA older adults^30^, ^31^ and cognitively unimpaired B/AA middle‐aged adults,^32^ compared to NHWs, and these cognitive changes in B/AA adults are associated with smaller changes in CSF tau.^32^ We previously reported baseline race‐related differences in Aβ and tau in cognitively unimpaired middle‐aged B/AA and NHW adults with a parental history of AD.^32^ In this study, we explored the relationship between CSF markers of vascular injury and neuroinflammation with established markers of disease pathology, and investigated racial differences in a diverse middle‐aged at‐risk cohort over a 2‐year follow‐up period.

## 2 METHODS

### 2.1 Study design and sample

The Association Between Cardiovascular Risk and Preclinical Alzheimer's Disease Pathology (ASCEND) Study was a 2‐year observational study of cognitively unimpaired or mildly impaired, middle‐aged B/AA and NHW adults at risk for AD due to parental history. Baseline results were reported previously.^32^ Briefly, we enrolled 82 middle‐aged (≥45 years) adults with a biological parent with either autopsy‐confirmed or probable AD as defined by National Institute of Neurological and Communicative Disorders and Stroke and Alzheimer's Disease and Related Disorders Association criteria^33^ and verified using the validated Dementia Questionnaire^34^ and medical records when available. Most participants were recruited from the Alzheimer's Disease Research Center, where the parent with AD underwent a full medical work‐up by a physician with expertise in dementia diagnoses.

Exclusion criteria included contraindication for lumbar puncture (LP); history of significant neurologic disease, head trauma, or major depression within the last 2 years; history of alcohol or substance abuse; diagnosis of AD, mild cognitive impairment (MCI), or residence in a skilled nursing facility; use of investigational medication; and unwillingness to fast. The Montreal Cognitive Assessment (MoCA) was used to assess cognitive impairment. MoCA scores >26 were considered normal, and scores between 18 and 25 were categorized as MCI.^35^ The ASCEND study included three annual visits (baseline, year 1, and year 2). Participants underwent LP at baseline and year 2 and a blood draw on all visits.

Medical history and sociodemographic information were collected via a self‐report questionnaire. As race is a social construct and collected by self‐report, terms and concepts around race and ethnicity are not universal. In the United States some individuals self‐identify race as Black, while others identify as African American. To be most inclusive, we are using both terms as they best reflect the racial identities of the study population.

**RESEARCH IN CONTEXT**

1. **Systematic review**: We reviewed the literature using traditional (e.g., PubMed, GoogleScholar) sources. Pathophysiologic mechanisms leading to the development of Alzheimer's disease (AD) often begin during mid‐life, especially among high‐risk individuals. Several pathologic mechanisms beyond amyloid and cholinergic processes are implicated in AD pathology, including vascular and renin angiotensin system dysfunction and inflammation. Although Black Americans have a higher incidence and prevalence of AD than non‐Hispanic White Americans, recent studies have found lower levels of cerebrospinal fluid (CSF) amyloid beta and tau. Identification of preclinical pathologic mechanisms leading to AD in racially diverse groups is urgently needed.
2. **Interpretation**: In a cohort of cognitively unimpaired middle‐aged adults with a parental history of AD, we found CSF AD markers were related to markers of vascular dysfunction, blood–brain barrier breakdown, and inflammation over 2 years. We also found evidence of changes in established AD markers over the 2‐year period, with differences by race remaining over time.
3. **Future directions**: Further work is warranted to better understand the role of cerebral vascular dysregulation in early pathologic changes leading to AD and how these changes may differ by race.

### 2.2 CSF and blood collection and analyses

After an 8 hour overnight fast, participants underwent LP to collect CSF for Aβ and tau, and markers of vascular dysfunction and inflammatory cytokines and chemokines, as previously described.^32^ Participants also underwent blood draw for analysis of (1) RAS function, including ACE activity; (2) plasma inflammatory markers; and (3) apolipoprotein E (*APOE*) genotyping.

CSF Aβ40, Aβ42, t‐tau, and p‐tau concentration were measured by Lumipulse technology^35^, ^36^ (Fujirebio). Cut‐offs for normal values were: Aβ1‐42 > 526 pg/mL; t‐tau < 409 pg/mL; p‐tau < 50.2 pg/mL; and Aβ42/40 < 0.072.^36^ CSF sPDGFRβ level was determined using a commercially available sandwich enzyme‐linked immunosorbent assay (Invitrogen Catalog # EHPDGFRB; ThermoFisher Scientific) following the manufacturer's protocol as previously described.^25^

Cytokines and chemokines (interleukin [I]L‐1α, IL‐1β, IL‐4, IL‐6, IL‐7, IL‐8, IL‐9, IL‐10, tumor necrosis factor alpha [TNF‐α], transforming growth factor alpha [TGF‐α], interferon [IFN]‐γ, TNF receptor [TNFR]1, TNFR2, macrophage‐derived chemokine [MDC], monocyte chemoattractant protein 1 [MCP‐1], and fractalkine [CX3CL1]) were measured in CSF and plasma using the MILLIPLEX MAP Human Cytokine/Chemokine Magnetic Bead Panel (HCYTOMAG‐60K; Merck‐Millipore). Endothelial injury markers (intercellular adhesion molecule 1 [ICAM‐1] and vascular cellular adhesion molecule 1 [VCAM‐1]) were measured using MILLIPLEX MAP Human Neurodegenerative Magnetic Bead Panel 3 (HNDG3MAG‐36K; Merck‐Millipore). C‐reactive protein (CRP) and serum amyloid protein (SAP) were measured using MILLIPLEX MAP Human Cardiovascular Disease Magnetic Bead Panel 3 (HCVD3MAG‐67K; Merck‐Millipore). Matrix metalloproteinase (MMP)‐1, MMP‐2, and MMP‐9 were measured using MILLIPLEX MAP Human MMP Magnetic Bead Panel 2 (HMMP2MAG‐55K; Merck‐Millipore). Tissue inhibitor of metalloproteinase (TIMP)‐1 and TIMP‐2 were measured using MILLIPLEX MAP Human TIMP Magnetic Bead Panel 1 (HTMP1MAG‐54K; Merck‐Millipore. All kits were run on the Luminex 200 platform. Assays were conducted following the manufacturer's protocol.

ACE1 activity was measured in CSF and serum using an ACE1‐specific fluorescence resonance energy transfer (FRET) peptide substrate (Abz‐FRK[Dnp]‐P; Enzo Life Sciences)^37^ and ACE2 activity was measured using the ACE2‐specific FRET substrate ([Mca‐APK][Dnp]; Enzo Life Sciences),^38^ as previously described.

*APOE* genotypes were used as a potential covariate and were determined by real‐time polymerase chain reaction using TaqMan SNP Genotyping Assays (Applied Biosystems Inc.) unique for each *APOE* single nucleotide polymorphism, rs429358 (Assay ID C 3084793 20) and rs7412 (Assay ID C 904973 10), according to manufacturer's protocol. Participants were not recruited based on *APOE* genotype.

### 2.3 Statistical analyses

Data normality of continuous variables was assessed by histogram and Shapiro–Wilk test. Demographic variables were summarized using descriptive statistics. Paired *t* test for normally distributed data and paired Wilcoxon signed rank test for non‐normally distributed data were used to compare change from baseline to year 2. Differences in categorical variables were analyzed by a chi‐square test. Differences for continuous variables between B/AAs and NHWs were compared using a two‐sample *t* test for normally distributed data and Mann‐Whitney *U* test for variables with non‐normal distribution. Effect sizes (ES) were calculated using Cohen *d*. Multiple linear regressions were used to investigate associations between ACE1 and ACE2 activity, CSF sPDGFRβ level, AD biomarkers, and markers of inflammation, with Bonferroni‐corrected *P*‐values. To control for confounding factors, age, sex, race, and comorbidities were included in all regression models. Associations between blood and CSF biomarkers were examined using Pearson correlations for normally distributed variables and Spearman correlations for variables with non‐normal distributions. Statistical analyses were performed by SAS version 9.4 (SAS Institute), with an alpha set at 0.05.

## 3 RESULTS

### 3.1 Demographics

Baseline demographic and clinical data were previously described.^32^ In brief, the mean age of participants was 59 ± 7 years, 38% identified as B/AA (*n* = 30), and 66% were female (*n* = 53). Medical history included high cholesterol (57%), high blood pressure (41%), and diabetes (2%). While 31% of participants reported having no history of the aforementioned vascular risk factors, 40% reported having one risk factor, 26% reported two risk factors, and 2% reported a history of all three (i.e., high cholesterol, hypertension, and diabetes). A higher proportion of B/AAs compared to NHWs had a history of hypertension (57% vs. 34%, respectively; *P* = 0.047). Forty‐seven percent (*n* = 38) were *APOE* ε4 positive (*n* = 9 with two alleles), with no differences by race (*P* = 0.883). MoCA scores ranged from 21 to 30, with 31% of participants scoring <26, suggestive of MCI.

### 3.2 Disease‐related changes in CSF AD biomarkers over time and according to race

**FIGURE 1:**
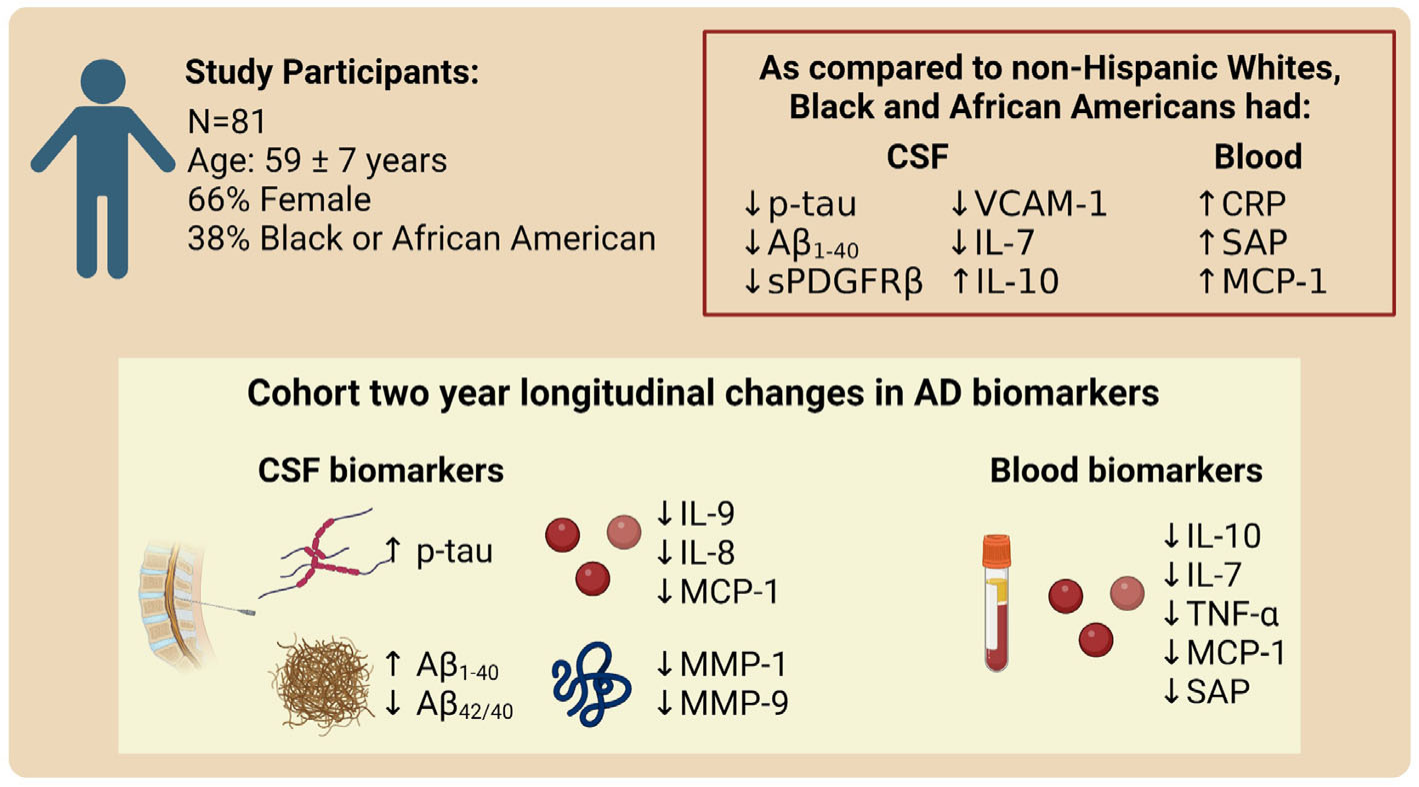
In a middle‐aged diverse cohort of persons with a parental history of AD, there were significant changes in AD CSF biomarkers over time, including increases in p‐tau and Aβ1‐4_0_. CSF AD biomarkers (p‐tau and Aβ1‐40) were lower among Black and African American participants compared to non‐Hispanic Whites. The CSF vascular markers sPDGFRβ and VCAM‐1 were also lower among Black and African American participants compared to non‐Hispanic Whites. Aβ, amyloid beta; AD, Alzheimer's disease; CRP, C‐reactive protein; CSF, cerebrospinal fluid; IL, interleukin; MCP, monocyte chemoattractant protein; MMP, matrix metalloproteinase; p‐tau, phosphorylated tau; SAP, serum amyloid protein; sPDGFRβ, soluble platelet‐derived growth factor beta; TNF, tumor necrosis factor; VCAM‐1, vascular cell adhesion molecule‐1.

We previously presented cross‐sectional baseline measurements of CSF Aβ and tau in these participants.^32^ Here, we present changes in CSF AD biomarkers over a subsequent 2‐year interval from baseline and in relation to race (Table 1); Figure 1 summarizes the major findings. Cut‐offs for normal values are based on those established by Gobom et al.^36^ as delineated in the Methods section.

**TABLE 1:** CSF biomarkers by race over 2 years.

	Total (*N* = 81)	Black/African American (*n* = 21)	Non‐Hispanic White (*n* = 46)	*P*‐value^*^	Time
**AD biomarkers**					
Aβ1‐40 (pg/mL)	9488.38 ± 2842.4 **10606.10 ± 3125** ^†^	8400.38 ± 2493.4 8546.94 ± 2305.2	9970.02 ± 2851.0 11372.30 ± 3061.3	**0.0336** **0.0014**	Baseline Year 2
Aβ1‐42 (pg/mL)	557.60 ± 242.7 517.44 ± 257.3	684.19 ± 200.8 656.13 ± 239.6	703.11 ± 261.3 744.05 ± 256.5	0.20 0.24	Baseline Year 2
Aβ42/40	0.07 (0.05–0.07) **0.06 (0.05–0.06)** ^†^	0.09 (0.08–0.09) **0.08 (0.07–0.09)** ^†^	0.08 (0.07–0.08) **0.07 (0.06–0.07)** ^†^	0.09 0.12	Baseline Year 2
p‐tau (pg/mL)	31.90 (32.3–43.9) **33.30 (35.3–49.9)** ^†^	25.00 (22.6–32.9) **26.90 (21.46–35.7)** ^†^	35.50 (33.5–45.7) **36.00 (36.90–53.3)** ^†^	**0.0103** **0.0046**	Baseline Year 2
t‐tau (pg/mL)	266.00 (264.5–354.8) 259.00 (265.3–363.9)	186.00 (165.7–244.1) 174.50 (134.8–294.7)	276.0 (278.8–374.1) 263.00 (276.8–379.9)	**0.0012** **0.0021**	Baseline Year 2
**Vascular markers**					
sPDGFRβ (pg/mL)	474.57 ± 157.9 462.14 ± 133.8	375.4 ± 151.6 378.9 ± 92.0	499.2 ± 166.4 480.6 ± 131.6	**0.0055** **0.0063**	Baseline Year 2
ICAM‐1 (pg/mL)	296.87 (295.7–398.4) 302.94 (289.5–412.6)	340.58 (268.45–463.57) 276.39 (227.2–424.0)	273.65 (278.3–389.78) 306.30 (291.2–429.8)	0.55 0.69	Baseline Year 2
VCAM‐1 (ng/mL)	25.27 (24.3–31.5) 27.10 (25.1–31.8)	20.40 (16.1–23.9) 18.50 (12.0–41.0)	27.16 (25.0–32.5) 28.38 (26.7–33.9)	**0.0040** **0.0094**	Baseline Year 2
ACE1 (nmol/min)	5562.74 ± 1683.8 5678.58 ± 1790.8	5325.52 ± 1053.4 5349.7 ± 1344.0	5753.34 ± 1768.8 5806.8 ± 1859.1	0.46 0.40	Baseline Year 2
ACE2 (nmol/min)	2172.28 ± 481.3 2268.09 ± 452.8	2245.69 ± 466.41 2291.2 ± 381.3	2203.82 ± 447.83 2277.5 ± 464.5	0.79 0.92	Baseline Year 2
**Cytokines and chemokines**					
IL‐10 (pg/mL)	7.66 ± 2.6 6.52 ± 1.9	7.16 ± 3.1 7.66 ± 5.2	7.11 ± 2.3 5.73 ± 2.0	0.79 **0.04**	Baseline Year 2
IL‐9 (pg/mL)	4.04 ± 1.3 **3.44 ± 1.5** ^†^	3.57 ± 1.4 3.12 ± 1.7	3.94 ± 1.6 3.69 ± 1.9	0.40 0.33	Baseline Year 2
IL‐7 (pg/mL)	3.12 ± 0.6 3.02 ± 1.5	2.59 ± 0.4 2.28 ± 1.2	3.04 ± 0.6 3.22 ± 2.2	**0.011** 0.17	Baseline Year 2
IL‐8 (pg/mL)	76.71 ± 20.4 **57.97 ± 12.2** ^†^	78.67 ± 14.1 70.53 ± 32.3	76.23 ± 20.6 53.52 ± 11.3	0.63 **0.004**	Baseline Year 2
TNFα (pg/mL)	2.90 ± 1.1 2.77 ± 0.6	2.98 ± 1.0 2.38 ± 0.9	2.93 ± 0.8 2.79 ± 1.0	0.86 0.16	Baseline Year 2
MCP‐1 (pg/mL)	6024.32 ± 556.7 **5290.64 ± 379.0** ^†^	6105.8 ± 659.1 5365.2 ± 333.4	5793.1 ± 756.2 5185.3 ± 440.6	0.11 0.14	Baseline Year 2
TGFα (pg/mL)	10.05 ± 1.5 –	10.11 ± 1.9 –	10.04 ± 2.16 –	0.90 –	Baseline Year 2
MDC (pg/mL)	127.52 ± 64.0 –	124.6 ± 75.0 –	50.82 ± 7.5 –	0.5 –	Baseline Year 2
Fractalkine (pg/mL)	– 135.58 ± 19.2	– 125.5 ± 23.9	– 132.6 ± 19.0	– 0.24	Baseline Year 2
**MMPs and TIMPs**					
MMP‐1 (pg/mL)	9.35 ± 6.7 **8.38 ± 5.7** ^†^	8.52 ± 6.4 8.57 ± 6.0	9.24 ± 6.1 8.48 ± 5.6	0.66 0.95	Baseline Year 2
MMP‐2 (ng/mL)	17.35 ± 4.0 16.48 ± 4.0	15.22 ± 6.1 15.21 ± 6.2	18.0.9 ± 5.7 16.53 ± 2.8	0.07 0.26	Baseline Year 2
MMP‐9 (pg/mL)	17.72 ± 11.9 **12.63 ± 8.3** ^†^	20.57 ± 17.0 12.71 ± 6.1	15.96 ± 6.6 12.90 ± 8.8	0.11 0.94	Baseline Year 2
TIMP‐1 (ng/mL)	36.54 ± 7.4 38.14 ± 12.0	36.63 ± 9.1 38.39 ± 20.8	36.75 ± 6.9 36.96 ± 6.0	0.95 0.68	Baseline Year 2
TIMP‐2 (ng/mL)	– 40.08 ± 8.8	– 37.91 ± 14.4	– 39.78 ± 5.8	– 0.48	Baseline Year 2
**TNF receptor superfamily**					
TNFR1 (pg/mL)	– 519.89 ± 158.0	– 464.4 ± 2007.5	– 541.7 ± 131.5	– 0.08	Baseline Year 2
TNFR2 (pg/mL)	– 789.26 ± 232.1	– 748.3 ± 299.7	– 804.5 ± 203.5	– 0.41	Baseline Year 2

*Note*: Results are reported as mean ± standard deviation or median (95% CI). Two‐sample *t* tests for normally distributed data and paired Wilcoxon signed rank test for non‐normally distributed data were used to test differences by race at each time point. Paired *t* tests for normally distributed data and paired Wilcoxon signed rank test for non‐normally distributed data were used to test differences by time and within each race by time.

Abbreviations: Aβ, amyloid beta; ACE, angiotensin converting enzyme; AD, Alzheimer's disease; CI, confidence interval; CSF, cerebrospinal fluid; ICAM, intercellular adhesion molecule; IL, interleukin; MCP, monocyte chemoattractant protein; MDC, macrophage‐derived chemokine; MMP, matrix metalloproteinase; p‐tau, phosphorylated tau; sPDGFRβ, soluble platelet‐derived growth factor receptor β; TGF, tumor growth factor; TIMP, tissue inhibitor matrix metalloproteinase; TNF, tumor necrosis factor; TNFR, tumor necrosis factor receptor; t‐tau, total tau; VCAM, vascular cell adhesion molecule.

* *P*‐values represent differences by race at each time point.

^†^ *P* < 0.05 versus baseline.

All bolded values are < 0.05

CSF levels of p‐tau increased from baseline to year 2, with moderate effect sizes for the cohort overall (ES = 0.600; *P* < 0.001), B/AAs (ES = 0.722; *P* = 0.022), and NHWs (ES = 0.626; *P* < 0.001). No differences over time for t‐tau were found. As previously reported,^32^ baseline CSF levels of p‐tau (ES = 0.650) and t‐tau (ES = 0.853) were lower among B/AA participants than NHW, and these differences remained at year 2 (ES = 0.693 and 0.697, respectively). CSF levels of Aβ_1‐40_ increased from baseline to year 2 (ES = 0.777; *P* < 0.001). As previously reported,^32^ baseline CSF levels of Aβ1‐40 were lower among B/AA participants than NHW (ES = 0.574) and these differences remained at year 2 (ES = 0.980). No differences by race were found in levels of Aβ1‐42. CSF levels of Aβ42/40 were significantly lower at year 2 compared to baseline for the cohort overall (ES = 0.587; *P* < 0.001), and for B/AAs (ES = 1.184; *P* = 0.002) and NHWs (ES = 0.722; *P* < 0.001).

Participants who were *APOE* ε4 positive (i.e., having at least one *APOE ε4* allele) had lower Aβ1‐42 (600 vs. 798 pg/mL; *P* = 0.001) and Aβ_42/40_ (0.068 vs. 0.084; *P* = 0.0008) compared to those without the ε4 allele. CSF levels of p‐tau and t‐tau did not differ by *APOE* ε4 allele when analyzed across the cohort or within groups by race. No associations between AD biomarkers and age or sex were found.

Median p‐tau and t‐tau levels were within normal range (<50.2 pg/mL and <409 pg/mL, respectively). However, p‐tau levels were higher than AD cut‐off limits in 12% of participants at baseline and 17% at year 2, while t‐tau levels were higher than AD cut‐off limits in 26% of participants at baseline and 27% at year 2.

While mean Aβ1‐42 was >526 pg/mL at baseline, the cut‐off for AD association^36^ adopted for this study, the number of participants with values lower than the cut‐off limits at baseline (35%) increased to 46% at year 2 (*P* < 0.001). Mean Aβ42/40 was 0.08 (95% confidence interval [CI] 0.07–0.08) at baseline and 0.07 (95% CI 0.06–0.07) at year 2 (ES = 0.60; *P* < 0.0001). A higher proportion of participants had a lower (< 0.072) Aβ42/40 at baseline compared to their year 2 measurements (76% vs. 93%; *P* = 0.013). Ten percent of participants had abnormal (i.e., resembling AD‐associated) levels of all three AT(N) AD biomarkers at both baseline and year 2.

### 3.3 CSF markers of vascular injury differed according to race and were related to markers of neuroinflammation and AD pathology

Mean levels of CSF markers of vascular injury and neuroinflammation at baseline and year 2 are listed in Table 1. There were no differences over time in vascular markers. When stratified according to race, CSF levels of sPDGFRβ were lower among B/AA participants compared to NHW at both baseline (ES = 0.720; *P* = 0.01) and year 2 (ES = 0.831; *P* = 0.006), with large effect sizes. In addition, CSF levels of VCAM‐1 were lower among B/AA participants compared to NHW at both baseline (ES = 0.734; *P* = 0.008) and year 2 (ES = 0.970; *P* = 0.002) with large effect sizes. No associations between the *APOE* ε4 allele and CSF levels of vascular markers were found.

CSF levels of the inflammatory cytokines IL‐9, IL‐8, and MCP‐1 were lower at year 2 compared to baseline (ES = 0.575, 0.648, and 0.973, respectively; *P* < 0.001). CSF IL‐10 (ES = 0.611; *P* = 0.04) and IL‐8 (ES = 0.885; *P* = 0.004) were significantly higher among B/AAs compared to NHWs at year 2. The matrix metalloproteinases MMP‐1 and MMP‐9 were significantly lower than baseline at year 2 (ES = 0.336 and 0.393, respectively; *P* ≤ 0.018). No other differences by race were found. At both baseline and year 2, individuals with at least one *APOE* ε4 allele had significantly lower levels of MMP‐2 (ES = 0.677 and 0.470 at year 2; *P* = 0.009) and TIMP‐1 (ES = 0.631 and 0.373; *P* ≤ 0.05) and higher levels of MMP‐9 (ES = 0.470 and 0.287; *P* ≤ 0.05).

**FIGURE 2:**
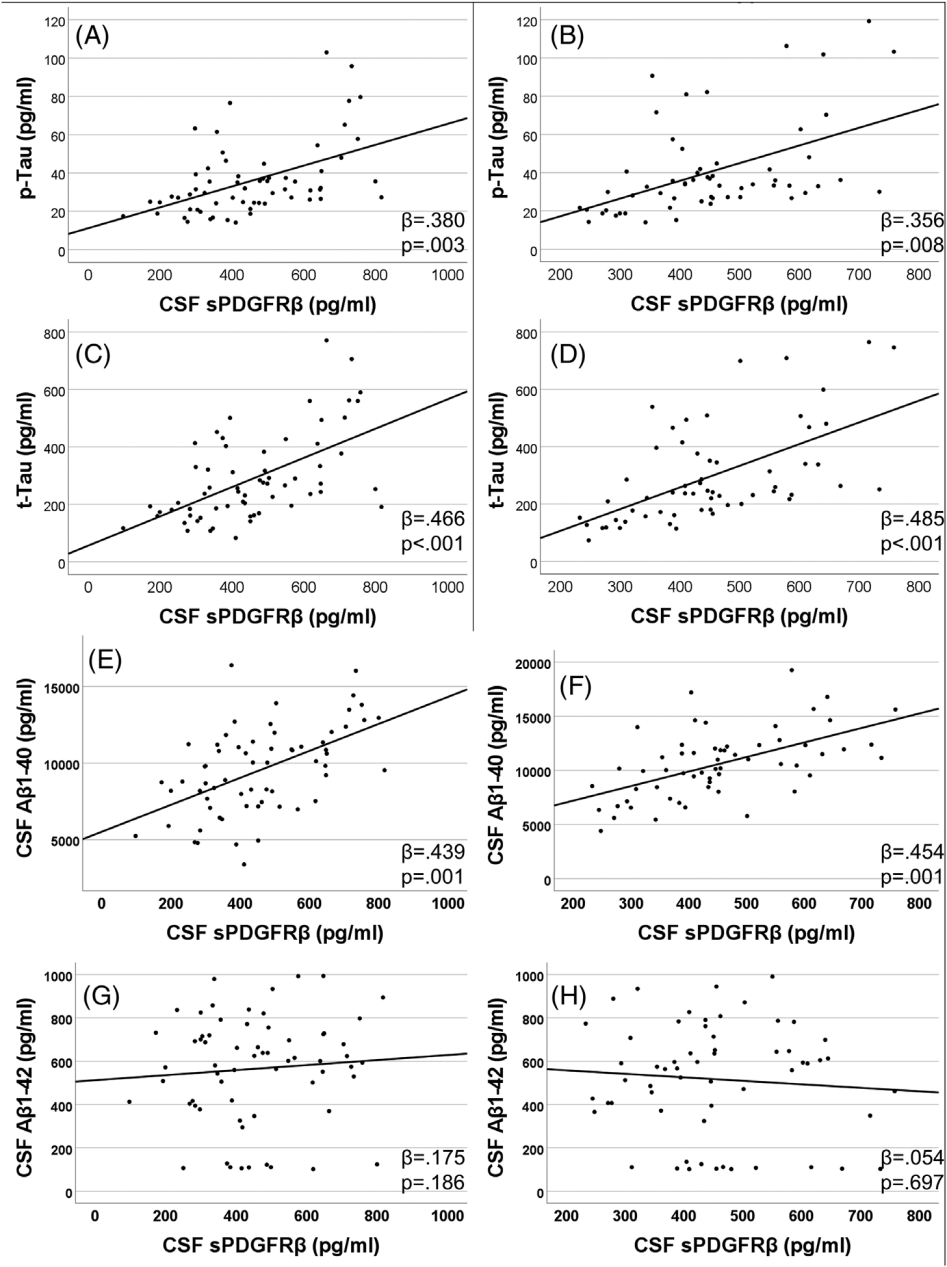
CSF sPDGFRβ was associated with CSF AD biomarkers over time. At both baseline and year 2, CSF sPDGFRβ was positively associated with CSF phosphorylated tau (A)–(B), total tau (C)–(D), Aβ peptide (E)–(F), and Aβ1–42 (G)–(H). Multiple linear analyses controlling for age, sex, race, and education were used. Aβ, amyloid beta; AD, Alzheimer's disease; CSF, cerebrospinal fluid; p‐tau, phosphorylated tau; sPDGFRβ, soluble platelet‐derived growth factor beta; t‐tau, total tau.

CSF sPDGFRβ level, a marker of pericyte injury, was positively associated with CSF p‐tau (β = 0.380; *P* = 0.003) and t‐tau (β = 0.466; *P* < 0.001) at baseline, and at year 2 follow‐up (β = 0.356; *P* = 0.008 and β = 0.485; *P* < 0.001, respectively) after controlling for age, sex, and race (Figure 2A–D). CSF sPDGFRβ was also positively related to CSF levels of Aβ1‐40 at baseline (β = 0.439; *P* = 0.001) and year 2 (β = 0.454; *P* = 0.001), after controlling for age, sex, and race (Figure 2E–F). No associations were observed between CSF sPDGFRβ and Aβ1‐42 at baseline or year 2 (Figure 2G–H). Lower than normal levels of Aβ1‐42 (i.e., <526 pg/mL) were associated with higher changes in sPDGFRβ (mean difference 62.3 pg/mL; *P* = 0.041; ES = 0.596). CSF sPDGFRβ was higher among individuals with abnormal AT(N) biomarkers compared to those with at least one biomarker within normal range at year 2 (581.2 ± 182 vs. 443.7 ± 122 pg/mL; *P* = 0.039; ES = 0.710).

**FIGURE 3:**
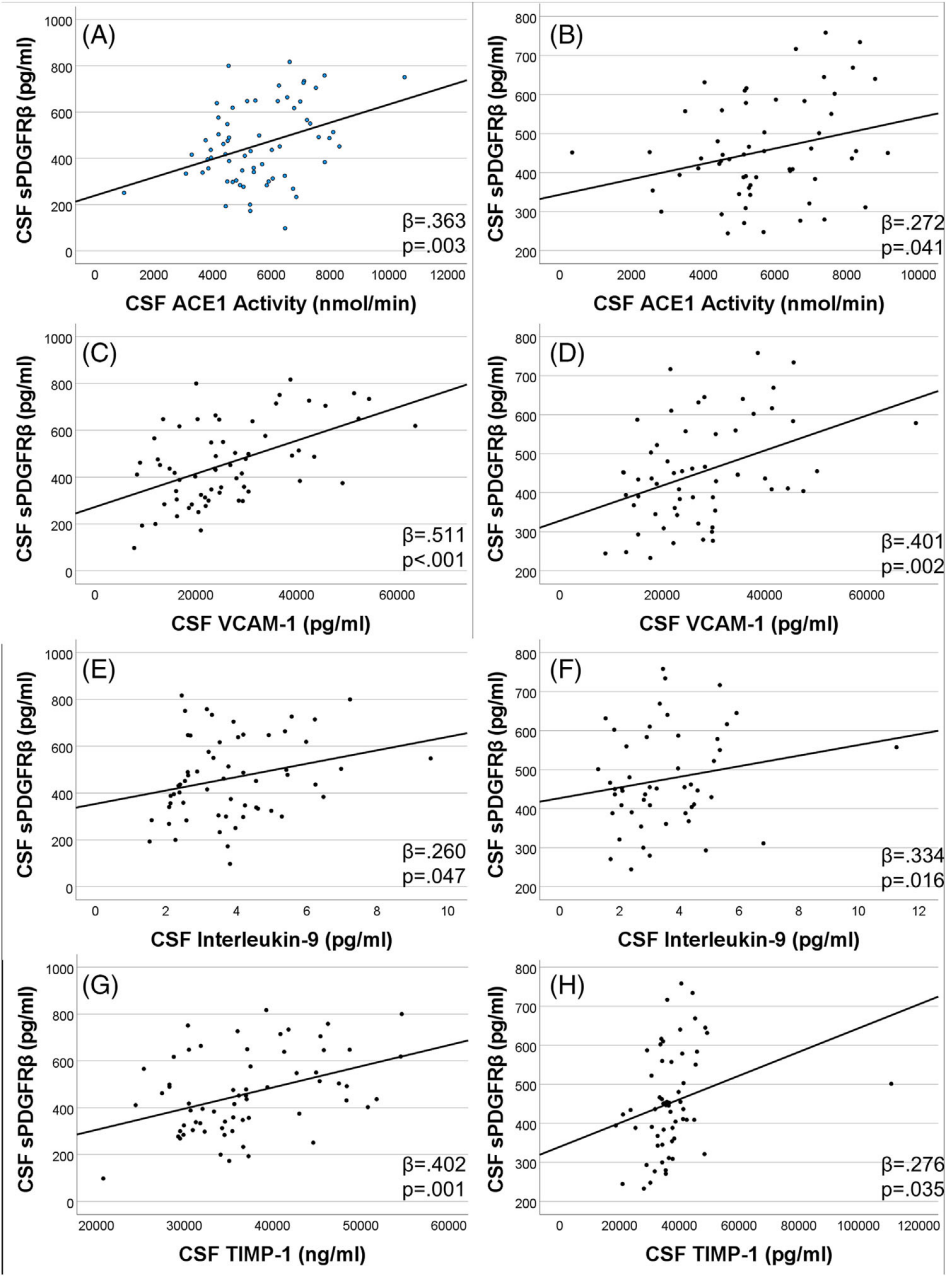
CSF sPDGFRβ was associated with CSF markers and mediators of vascular endothelial injury over time. At both baseline and year 2, CSF sPDGFRβ was positively associated with CSF ACE‐1 activity (A)–(B), and VCAM‐1 (C)–(D), IL‐9 (E)–(F), and TIMP‐1 (G)–(H). Multiple linear analyses controlling for age, sex, race, and education were used. ACE, angiotensin‐converting enzyme; CSF, cerebrospinal fluid; IL, interleukin; sPDGFRβ, soluble platelet‐derived growth factor beta; TIMP, tissue inhibitor of metalloproteinase; VCAM, vascular cell adhesion molecule.

**FIGURE 4:**
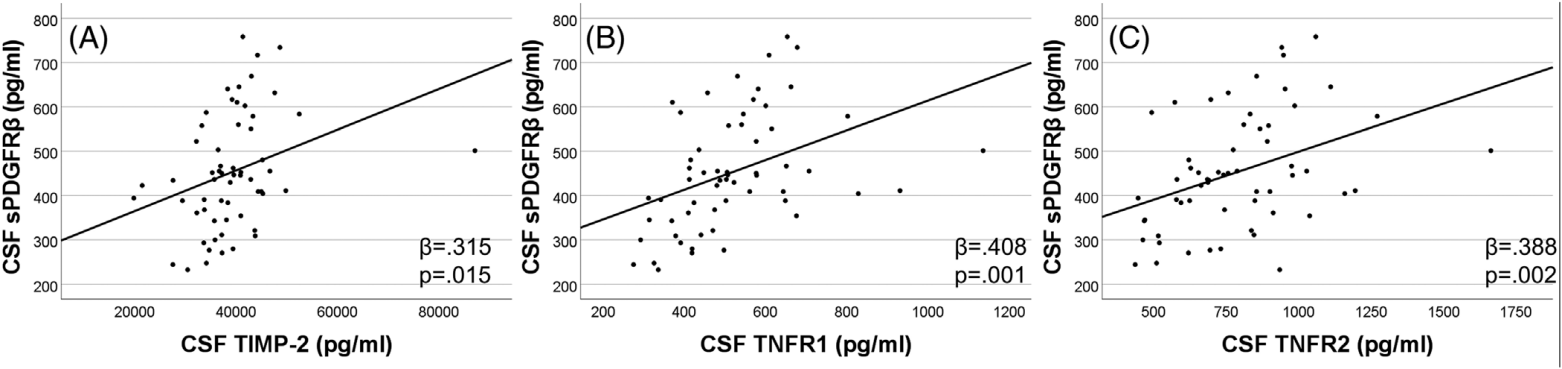
CSF sPDGFRβ was associated with CSF mediators of vascular endothelial injury at year 2. CSF sPDGFRβ was positively associated with CSF TIMP 2 (A), TNFR1 (B), and TNFR2 (C). These measures were not analyzed at baseline. Multiple linear analyses controlling for age, sex, race, and education were used. CSF, cerebrospinal fluid; IL, interleukin; sPDGFRβ, soluble platelet‐derived growth factor beta; TIMP, tissue inhibitor of metalloproteinase; TNFR, tumor necrosis factor receptor; VCAM‐1, vascular cell adhesion molecule‐1.

CSF sPDGFRβ was positively associated with markers and mediators of vascular endothelial injury: VCAM‐1 (β = 0.51; *P* < 0.0001), ACE‐1 activity (β = 0.36; *P* = 0.0032), TIMP‐1 (β = 0.40; *P* = 0.001), and MMP‐2 (β = 0.46; *P* = 0.0001) at baseline (Figure 3A,C,E). These associations remained at year 2 (Figure 3B,D,F). CSF sPDGFRβ was positively associated with IL‐9 at baseline and year 2 (β = 0.260; *P* = 0.047 and β = 0.334; *P* = 0.016; Figure 3G–H). Additionally, CSF sPDGFRβ positively related to TIMP‐2 (β = 0.32; *P* = 0.015), TNFR‐1 (β = 0.41; *P* = 0.0013), and TNFR‐2 (β = 0.39; *P* = 0.0024) at year 2 (Figure 4A–C); these measures were not assessed at year 1.

### 3.4 Circulating levels of vascular markers remain relatively stable

Plasma and serum vascular and inflammatory biomarkers are listed in Table 2. There were no differences in circulating vascular markers from baseline to year 2; however, VCAM‐1 was significantly higher at year 1 than baseline (ES = 0.300; *P* = 0.016). VCAM‐1 was significantly lower among B/AAs compared to NHWs at baseline (ES = 0.530) and year 1 (Cohen *d* = 0.283), with no differences at year 2.

**TABLE 2 alz13457-tbl-0002:** Plasma/serum biomarkers by race over 2 years.

	Total (*N* = 81)	Black/African American (*N* = 21)	Non‐Hispanic White (*N* = 46)	*P*‐value^*^	Time
**Vascular biomarkers**					
ICAM‐1 (ng/mL)	138.02 (125.6–168.8) 132.48 (122.2–150.8) 123.94 (114.5–138.5)	136.85 (129.6–148.8) 137.61 (111.4–151.0) 135.11 (113.8–151.2)	137.20 (122.7–190.0) 129.53 (119.8–158.2) 123.98 (112.7–136.5)	0.75 0.87 0.32	Baseline Year 1 Year 2
VCAM‐1 (ng/mL)	842.95 (760.2–953.8) **891.06 (955.8–1741.18)** ^†^ 866.51 (852.4–1729.9)	807.55 (614.7–883.3) 746.59 (504.6–1581.6) 827.73 (0–3827.3)	863.46 (808.4–1074.2) 930.16 (963.3–2011.5) 896.74 (1028.6–2828.8)	**0.02** **0.03** 0.32	Baseline Year 1 Year 2
ACE1 (nmol/min)	24121.97 ± 10953.8 25072.49 ± 13399.1 24599.05 ± 12979.2	23160.0 ± 11818.4 22151.4 ± 12306.5 24576.4 ± 12446.7	25636.0 ± 10422.7 25916.7 ± 13893.9 25259.9 ± 11153.1	0.36 0.59 0.82	Baseline Year 1 Year 2
ACE2 (nmol/min)	1501.74 ± 963.3 1511.97 ± 1044.8 1479.92 ± 1038.5	1273.6 ± 924.7 1223.8 ± 772.4 1416.2 ± 968.6	1554.8 ± 893.4 1629.6 ± 1127.5 1584.5 ± 1152.2	0.21 0.09 0.55	Baseline Year 1 Year 2
**Cytokines and chemokines**					
IL‐10 (pg/mL)	11.30 ± 8.6 **9.29 ± 4.5** ^†^ **9.37 ± 4.1** ^†^	9.23 ± 5.0 8.75 ± 4.28 9.23 ± 5.1	12.09 ± 8.5 **9.53 ± 4.7** ^†^ **9.75 ± 0.7** ^†^	0.09 0.53 0.68	Baseline Year 1 Year 2
IL‐9 (pg/mL)	1.64 (0–38.8) 4.44 (4.7–11.6) 4.71 (4.8–10.6)	4.16 (0–72.2) 5.66 (3.1–10.3) 5.62 (2.1–10.5)	4.67 (0–65.0) 3.52 (3.84–13.8) 4.52 (4.3–11.8)	0.94 0.83 0.72	Baseline Year 1 Year 2
IL‐7 (pg/mL)	5.38 ± 2.2 **4.64 ± 1.9** ^†^ **4.94 ± 2.0** ^‡^	5.98 ± 2.5 5.51 ± 2.5 5.19 ± 2.0	4.86 ± 1.6 **4.28 ± 1.5** ^†^ **4.99 ± 2.3** ^‡^	**0.03** **0.02** 0.71	Baseline Year 1 Year 2
IL‐8 (pg/mL)	5.36 (10.8–21.3) 5.10 (8.8–26.5) 4.59 (8.8–22.6)	4.28 (4.7–14.9) 4.15 (5.3–20.4) 3.51 (4.6–16.0)	7.03 (12.0–29.0) 5.29 (7.2–32.6) 4.94 (8.9–27.6)	0.08 0.42 0.19	Baseline Year 1 Year 2
TNFα (pg/mL)	6.70 ± 3.2 **6.02 ± 3.0** ^†^ **5.91 ± 3.0** ^†^	6.33 ± 1.7 **5.54 ± 1.8** ^†^ **6.0 ± 1.1** ^†^	6.68 ± 3.7 6.25 ± 3.34 6.1 ± 3.5	0.58 0.38 0.92	Baseline Year 1 Year 2
MCP1 (pg/mL)	198.00 ± 81.3 198.05 ± 88.3 **171.94 ± 59.8** ^†^	224.4 ± 84.4 203.26 ± 65.1 196.4 ± 49.8	176.8 ± 74.4 195.67 ± 97.6 **165.4 ± 9.7** ^‡^	**0.002** 0.75 **0.05**	Baseline Year 1 Year 2
TGFα (pg/mL)	3.4 (2.9–13.0) – –	3.47 (0–27.3) – –	2.75 (2.44–9.96) – –	0.41 – –	Baseline Year 1 Year 2
IFNγ (pg/mL)	7.42 (0–1077.3) 6.74 (7.3–21.1) 7.15 (7.0–14.8)	7.99 (0–657.0) 6.16 (0.2–34.0) 5.92 (5.4–16.8)	9.61 (0–1523.1) 6.75 (6.0–19.6) 6.82 (5.5–16.9)	0.43 0.91 0.78	Baseline Year 1 Year 2
CRP (pg/mL)	7.16 (7.5–15.3) 6.93 (3.3–27.7) 5.43 (3.3–47.7)	13.17 (4.2–50.4) 10.95 (6.3–20.6) 10.49 (8.4–19.7)	4.94 (5.0–9.2) 5.18 (0–34.3) 4.77 (6.0–10.1)	**0.002** 0.084 **0.03**	Baseline Year 1 Year 2
SAP (pg/mL)	9.57 ± 3.5 **8.64 ± 2.8** ^†^ **8.65 ± 3.2** ^†^	10.09 ± 4.5 9.55 ± 3.0 9.99 ± 2.7	8.87 ± 2.5 **8.23 ± 2.7** ^†^ 8.39 ± 3.5	**0.04** **0.03** **0.04**	Baseline Year 1 Year 2
MDC (pg/mL)	1163.42 ± 533.9 1149.00 ± 353.4 1171.80 ± 441.3	1373.3 ± 627.0 1173.98 ± 403.7 1281.7 ± 540.1	1061.8 ± 430.4 1137.64 ± 332.4 1100.8 ± 384.4	**0.006** 0.71 0.11	Baseline Year 1 Year 2
Fractalkine (pg/mL)	– 163.06 ± 58.9 170.43 ± 54.8	185.3 ± 25.5 183.09 ± 84.5 178.2 ± 69.7	155.9 ± 57.7 153.96 ± 40.7 167.2 ± 51.9	0.24 0.16 0.51	Baseline Year 1 Year 2
IL‐6 (pg/mL)	– 4.75 (10.7–35.3) 4.36 (11.3–31.8)	– 3.44 (4.3–27.4) 2.44 (0–27.4)	– 5.04 (8.8–44.2) 7.69 (10.6–40.8)	– 0.55 0.18	Baseline Year 1 Year 2
IL‐1α (pg/mL)	– 30.23 (21.3–60.3) 15.37 (17.9–55.0)	– 16.15 (6.8–57.5) 23.25 (0–111.5)	– 20.23 (17.5–72.2) 17.98 (18.7–49.8)	– 0.87 0.60	Baseline Year 1 Year 2
IL‐1β (pg/mL)	– 2.98 ± 1.4 2.73 ± 125	– 2.80 ± 1.1 2.55 ± 1.2	– 3.10 ± 1.5 2.95 ± 1.3	– 0.56 0.41	Baseline Year 1 Year 2
IL‐2 (pg/mL)	– 2.33 ± 1.7 2.72 ± 1.8	– 1.85 ± 0.6 2.61 ± 0.3	– 2.67 ± 2.2 3.38 ± 2.4	– 0.37 0.43	Baseline Year 1 Year 2
IL‐4 (pg/mL)	– 58.69 (161.5–514.3) 71.75 (172.0–495.9)	– 53.99 (52.3–464.3) 49.53 (0–363.0)	– 63.83 (130.7–647.5) 88.8 (163.4–621.4)	– 0.60 0.08	Baseline Year 1 Year 2
IP‐10 (pg/mL)	– 304.13 ± 307.0 266.83 ± 200.1	– 230.39 ± 104.1 242.3 ± 79.9	– 337.65 ± 360.1 **254.6 ± 131.9** ^‡^	– 0.07 0.66	Baseline Year 1 Year 2
**TNF receptor superfamily**					
TNFR1 (pg/mL)		– 128.81 ± 77.9 110.3 ± 57.8	– 126.89 ± 76.6 122.6 ± 61.7	– 0.93 0.48	Baseline Year 1 Year 2
TNFR2 (pg/mL)		– 922.93 ± 294.5 928.3 ± 270.8	– 927.92 ± 350.9 891.1 ± 210.5	– 0.96 0.57	Baseline Year 1 Year 2

*Note*: Results are reported as mean ± standard deviation or median (95% CI). Two‐sample t‐tests for normally distributed data and paired Wilcoxon signed rank test for non‐normally distributed data were used to test differences by race at each time point. Least squares mean analyses were used to test differences over time, differences by race at each time point.

Abbreviations: ACE, angiotensin‐converting enzyme; CI, confidence interval; CRP, C‐reactive protein; ICAM, intercellular adhesion molecule; IFN, interferon; IL, interleukin; IP, interferon gamma‐induced protein; MCP, monocyte chemoattractant protein; MDC, macrophage‐derived chemokine; MMP, matrix metalloproteinase; SAP, serum amyloid protein; TGF, tumor growth factor; TNF, tumor necrosis factor; TNFR, tumor necrosis factor receptor; VCAM, vascular cell adhesion molecule.

* *P*‐values represent differences by race at each time point.

^†^ *P* < 0.05 versus baseline.

^‡^ *P* < 0.05 versus year 1.

All bolded values are < 0.05

### 3.5 Circulating levels of markers of neuroinflammation decreased over time

IL‐10 was significantly lower than baseline at year 1 (ES = 0.309; *P* = 0.0043) and year 2 (ES = 0.325; *P* = 0.0058). No significant differences between year 1 and year 2 were found. Among NHWs, IL‐10 was significantly lower than baseline at year 1 (ES = 0.315; *P* = 0.0072) and year 2 (ES = 0.332; *P* = 0.0135), with no significant differences between year 1 and year 2. No differences over time among B/AAs were found.

IL‐7 was significantly lower at year 1 than baseline (ES = 0.359; *P* = 0.0037), and year 2 was significantly lower than year 1 (ES = 0.291; *P* = 0.0135). No significant differences between baseline and year 2 were found. Among NHWs, IL‐7 was significantly lower at year 1 compared to baseline (ES = 0.438; *P* = 0.029), and year 2 was significantly higher than year 1 (ES = 0.319; *P* = 0.022), with no differences between baseline and year 2. No differences over time among B/AAs were found. IL‐7 was significantly higher among B/AAs compared to NHWs at baseline (ES = 0.541) and year 1 (ES = 0.656), with no differences at year 2.

TNF‐α was significantly lower than baseline at year 1 (ES = 0.283; *P* = 0.020) and year 2 (ES = 0.320; *P* = 0.008). No differences between year 1 and year 2 were found. Among B/AAs, TNF‐α was significantly lower than baseline at year 1 (ES = 0.561; *P* = 0.011) and year 2 (ES = 0.556; *P* = 0.010), with no differences between years 1 and 2. No differences over time between NHWs were found.

MCP‐1 was significantly lower at year 2 compared to baseline (ES = 0.365; *P* = 0.0009) and year 1 (ES = 0.441; *P* = 0.0009). MCP‐1 was significantly lower at year 2 compared to year 1 among B/AAs (ES = 0.458; *P* = 0.005) and compared to baseline among NHWs (ES = 0.620; *P* = 0.010). MCP‐1 was significantly higher among B/AAs compared to NHWs at baseline (ES = 0.774) and year 2 (ES = 0.420).

SAP was significantly lower at year 1 (ES = 0.284; *P* = 0.0126) and year 2 (ES = 0.233; *P* = 0.0472) compared to baseline. Among NHWs, SAP was significantly lower than baseline at year 1 (ES = 0.366; *P* = 0.300), with no significant differences in year 2. No differences between year 1 and year 2 were found. SAP was significantly higher among B/AAs compared to NHWs at all three time points (ES = 0.421, 0.475, and 0.416, respectively).

There were no significant differences over time for CRP, a marker of general systemic inflammation. However, CRP was significantly elevated (2–3‐fold increase) among B/AA participants compared to NHW at both baseline and year 2, with medium to large effect sizes (ES = 0.757 and 0.442, respectively). IP‐10 was collected at year 1 and year 2 only. No differences over time were found among the cohort; however, IP‐10 was significantly lower at year 2 compared to year 1 among NHWs (ES = 0.290; *P* = 0.0272).

CRP was significantly lower among individuals with at least one copy of the *APOE* ε4 allele at baseline (3.91 vs. 9.24 pg/mL; ES = 0.405; *P* = 0.013), year 1 (4.75 vs. 8.62 pg/mL; ES = 0.127; *P* = 0.042), and year 2 (4.11 vs. 9.19 pg/mL; ES = 0.341; *P* = 0.017). SAP was significantly lower among individuals with at least one copy of the *APOE* ε4 allele at baseline (8.66 vs. 10.34 pg/mL; ES = 0.491; *P* = 0.038) and year 2 (7.65 vs. 9.34 pg/mL; ES = 0.552; *P* = 0.024).

CSF TNF‐α was positively associated with levels of plasma TNF‐α at baseline (*r* = 0.405; *P* = 0.021), with no association at year 2. CSF levels of VCAM‐1 were positively associated with plasma VCAM‐1 at both baseline (rho = 0.329; *P* = 0.029) and year 2 (rho = 0.330; *P* = 0.035). No associations between CSF and blood levels of other variables were found.[Table alz13457-tbl-0002]

## 4 DISCUSSION

Here we report longitudinal data from a 2‐year follow‐up period of previously reported baseline results from ASCEND in cognitively unimpaired or mildly impaired B/AA and NHW middle‐aged adults with a parental history of AD.^32^ Our data demonstrate changes in established AD biomarkers with moderate to large effect sizes, consistent with early disease manifestations. We also highlight significant race‐related differences in disease‐related and vascular markers within the cohort that require further investigation.

In this cohort of at‐risk middle‐aged adults, we found changes consistent with increased risk in CSF AD biomarkers between baseline and year 2. While median CSF t‐tau remained within normal limits over the 2‐year period, there was a significant increase in p‐tau from baseline to year 2. CSF Aβ42/40 decreased and Aβ1‐40 increased over 2 years. Although Aβ1‐40 is less frequently discussed as a biomarker of AD, several studies have found higher CSF Aβ1‐40 in MCI, prodromal AD, and AD;^39^, ^40^, ^41^, ^42^ however, other studies found no differences with progression to AD.^43^, ^44^, ^45^, ^46^ As this cohort is middle aged with low to moderate vascular risk factors, further work is needed to better understand the contribution of Aβ1‐40 to AD risk.

Half of participants at baseline and three fifths of participants at year 2 had below‐normal Aβ1‐42 levels, indicative of a worsening AD profile. Lower CSF Aβ1‐42 is considered one of the earliest indicators of AD neuropathologic change.^47^, ^48^ As these findings suggest the higher AD risk inferred by having a first‐degree relative with AD may be associated with higher amyloid burden, a more comprehensive understanding of the interplay of family history, genetics, and vascular risk factors among high‐risk populations is urgently needed. Ten percent of ASCEND participants met the definition for AD with respect to AT(N) biomarker levels.^2^ Together, these data may suggest AD pathological changes in this middle‐aged, at‐risk cohort, although this cohort is unlikely to have significant evidence of disease. While this cohort has an increased dementia risk due to parental history, the development of dementia is not inevitable. Approximately half of participants in this study were *APOE* ε4 positive, consistent with other cohorts of adult children of a parent with an AD diagnosis. However, it is important to note we cannot rule out the possibility of parental history of vascular or mixed dementia. We are continuing to follow these individuals over time to better understand the progression of AD biomarkers in high‐risk, diverse cohorts.

No changes in CSF vascular markers over 2 years were found. There were decreases in CSF and plasma markers of inflammation and CSF MMPs from baseline to year 2, which was unexpected considering increasing age and vascular comorbidities are associated with higher vascular dysfunction and inflammation. Changes in these markers over time were not associated with age or comorbidities. Decreases in inflammation could be a product of participation in a research study, as participants had access to education events, which included information on the role of inflammation and risk reduction.

Our baseline findings were the first to report lower levels of t‐tau and p‐tau based on race in middle‐aged adults with an increased risk for AD.^32^ We now demonstrate tau levels remained lower among B/AA compared to NHW over a 2‐year period, consistent with recent analyses of racial differences in AD biomarkers in a population with cognitive impairment.^31^, ^49^ We also found that CSF markers of vascular dysfunction were lower in B/AA compared to NHW at baseline and year 2. This supports a recent study in which cardiovascular comorbidities were not associated with CSF tau levels in B/AA participants.^49^ No association with *APOE* ε4 was found in this study; this might be explained by the cognitively unimpaired or MCI composition of this cohort.^50^

If there are differences in AD biomarkers by race, it will be increasingly important to understand appropriate cut‐offs for diagnosis and treatments. As potential treatments under development target tau and Aβ, it is important to establish whether certain treatments may be less effective for some communities. Within the research context, there is emerging evidence for differences by race in CSF biomarkers. However, there is insufficient biological data, especially CSF, in racial and ethnic minorities.^51^ More work is needed globally to better understand these emerging differences along with drivers of AD pathophysiologic processes in diverse populations.

Circulating inflammatory biomarkers were higher among B/AAs compared to NHWs, aligning with other research finding higher systemic inflammation among B/AAs compared to NHWs.^52^, ^53^, ^54^, ^55^, ^56^, ^57^ Higher chronic inflammation among B/AAs has been linked to effects of systemic racism and discrimination, likely due to increased activation of cortisol pathways^56^, ^58^, ^59^ and is thought to be a risk factor for cognitive changes and performance on cognitive function assessments in B/AA adults.^53^, ^60^ However, few studies consider the potential effects of predisposing psychosocial factors, such as stress and discrimination, on the relationship between inflammation and cognitive function. Further work is needed to understand the role of peripheral and central processes in AD risk among diverse populations.

In this cohort of middle‐aged adults with a parental history of AD, 36% of participants had one *APOE* ε4 allele and 11% were homozygous, higher than the general population (9%–23% and 2%–3%, respectively).^61^ The frequency of *APOE* ε4 genotype is generally higher among B/AAs (19%) compared to NHWs (14%),^61^ although there were no differences in this study. CSF Aβ42 and Aβ42/40 were significantly lower in persons with at least one *APOE* ε4 allele, which may relate to additional AD risk in persons with a parent with AD. While *APOE* ε4 genotype confers an increased genetic risk factor, not all persons who are ε4 positive develop AD, even with a family history. Considering other known risk factors, many of which are vascular, other possible outcomes include the development of vascular dementia or a mixed phenotype.

We also report associations between CSF markers of vascular injury and neuroinflammation in relation to markers of established disease pathology, indicative of early disease changes. CSF sPDGFRβ was positively associated with markers and mediators of vascular injury and brain cytokines and correlated with disease pathology. Interestingly, CSF sPDGFRβ, ICAM‐1, and ACE‐1 were lower in B/AA compared to NHW for reasons that are not yet clear. These findings are surprising considering the higher proportion of B/AAs with a history of hypertension compared to NHWs in this cohort. These data suggest vascular injury and neuroinflammation are related to early changes in AD pathology in a cognitively unimpaired or mildly impaired middle‐aged at‐risk cohort.

Markers of vascular injury, particularly sPDGFRβ, were strongly associated with levels of p‐tau, t‐tau, and Aβ1‐40, but surprisingly not Aβ1‐42. However, persons with low Aβ1‐42 had a larger increase in sPDGFRβ compared to individuals with normal Aβ1‐42, supporting a previous study showing CSF sPDGFRβ level correlated with t‐tau and p‐tau in AD.^25^ These data suggest pericyte dysfunction occurs early in the disease process, but further studies are required to confirm whether CSF sPDGFRβ changes are related to, or independent from, changes in CSF tau and Aβ.

Our data indicate sPDGFRβ is associated with markers of vascular endothelial activation, mediators of endothelial dysfunction, and IL‐9. These findings support previous observations linking vascular dysfunction and central nervous system inflammation in the early pre‐symptomatic phases of dementia. In a study of persons with MCI and AD, raised CSF levels of vascular markers and cytokines were associated with t‐tau and p‐tau, cortical thinning, and poorer Mini‐Mental State Examination scores; the associations were stronger in Aβ‐positive individuals.^11^ Pericyte loss and BBB breakdown have been associated with elevated levels of brain cytokines in early stages of AD,^62^ and serum markers of vascular injury and inflammation increased over time with a decline in MCI compared to those that remained MCI‐stable.^63^

Our findings identified mediators of vascular homoeostasis, including MMP‐2 and TIMP‐1 and TIMP‐2 were positively correlated with sPDGFRβ, supporting other work suggesting TNFR dysregulation may be associated with tau pathology and may play a neurodegenerative role in AD pathology.^64^, ^65^ It is important to note that in a sample size of 81, we are unable to rule out other factors that may mediate relationships between sPDGFRβ and other CSF markers of AD pathology. Further work in larger studies with diverse populations is needed to better understand the role of sPDGFRβ in early AD pathology.

In conclusion, in a cohort of cognitively unimpaired or mildly impaired middle‐aged adults at risk of developing AD, we found evidence of changes in established CSF biomarkers at 2‐year follow‐up consistent with early changes in the development of AD (CSF t‐tau/p‐tau and Aβ40). CSF markers of vascular dysfunction, including pericyte damage and endothelial injury, were associated with changes in MMP‐2 and TIMPs, and cytokines. Elevated sPDGFRβ was related to t‐tau, p‐tau, and changes in Aβ40 but not Aβ42. Interstitial fluid tau, sPDGFRβ, ACE‐1, and ICAM‐1 were lower in B/AA compared to NHW, which is worth noting with respect to what are perceived normative ranges for patients of different racial backgrounds. These data indicate that cerebral vascular dysregulation may be a very early event that occurs in the development of AD, detectable in a mid‐life cognitively unimpaired or mildly impaired cohort, and that race impacts these relationships, warranting further research.

## CONFLICT OF INTEREST STATEMENT

H.Z. has served on scientific advisory boards and/or as a consultant for Abbvie, Acumen, Alector, ALZPath, Annexon, Apellis, Artery Therapeutics, AZTherapies, CogRx, Denali, Eisai, Nervgen, Novo Nordisk, Passage Bio, Pinteon Therapeutics, Red Abbey Labs, reMYND, Roche, Samumed, Siemens Healthineers, Triplet Therapeutics, and Wave; has given lectures in symposia sponsored by Cellectricon, Fujirebio, Alzecure, Biogen, and Roche; and is a co‐founder of Brain Biomarker Solutions in Gothenburg AB (BBS), which is a part of the GU Ventures Incubator Program (outside submitted work). B.B., H.H., W.T.H., P.G.K., J.S.M., D.D.V., L.Z., and W.W. have nothing to disclose. Author disclosures are available in the supporting information.

## CONSENT STATEMENT

Each subject was informed of the testing protocols and potential risks and benefits of participation. All participants provided written informed consent before participation.

## Supporting information

Supporting Information

## References

[1] Sperling RA , Aisen PS , Beckett LA , et al. Toward defining the preclinical stages of Alzheimer's disease: recommendations from the National Institute on Aging‐Alzheimer's Association workgroups on diagnostic guidelines for Alzheimer's disease. Alzheimers Dement. 2011;7(3):280‐292. doi:10.1016/j.jalz.2011.03.003 21514248 PMC3220946

[2] Jack CR , Bennett DA , Blennow K , et al. NIA‐AA research framework: toward a biological definition of Alzheimer's disease. Alzheimers Dement. 2018;14(4):535‐562. doi:10.1016/j.jalz.2018.02.018 29653606 PMC5958625

[3] Selkoe DJ , Hardy J . The amyloid hypothesis of Alzheimer's disease at 25 years. EMBO Mol Med. 2016;8(6):595‐608. 10.15252/emmm.201606210 27025652 PMC4888851

[4] Bartus RT , Dean RL 3rd , Beer B , Lippa AS . The cholinergic hypothesis of geriatric memory dysfunction. Science. 1982;217(4558):408‐414. doi:10.1126/science.7046051 7046051

[5] de la Torre JC . The vascular hypothesis of Alzheimer's disease: bench to bedside and beyond. Neurodegener Dis. 2010;7(1‐3):116‐121. doi:10.1159/000285520 20173340

[6] Kehoe PG . The coming of age of the angiotensin hypothesis in Alzheimer's disease: progress toward disease prevention and treatment? J Alzheimers Dis. 2018;62(3):1443‐1466. doi:10.3233/jad-171119 29562545 PMC5870007

[7] McGeer PL , McGeer EG . The amyloid cascade‐inflammatory hypothesis of Alzheimer disease: implications for therapy. Acta Neuropathol. 2013;126(4):479‐497. doi:10.1007/s00401-013-1177-7 24052108

[8] Sweeney MD , Montagne A , Sagare AP , et al. Vascular dysfunction‐The disregarded partner of Alzheimer's disease. Alzheimers Dement. 2019;15(1):158‐167. doi:10.1016/j.jalz.2018.07.222 30642436 PMC6338083

[9] Korte N , Nortley R , Attwell D . Cerebral blood flow decrease as an early pathological mechanism in Alzheimer's disease. Acta Neuropathol. 2020;140(6):793‐810. doi:10.1007/s00401-020-02215-w 32865691 PMC7666276

[10] Frohman EM , Frohman TC , Gupta S , de Fougerolles A , van den Noort S . Expression of intercellular adhesion molecule 1 (ICAM‐1) in Alzheimer's disease. J Neurol Sci. 1991;106(1):105‐111. doi:10.1016/0022-510x(91)90202-i 1685745

[11] Janelidze S , Mattsson N , Stomrud E , et al. CSF biomarkers of neuroinflammation and cerebrovascular dysfunction in early Alzheimer disease. Neurology. 2018;91(9):e867. doi:10.1212/wnl.0000000000006082 30054439 PMC6133624

[12] Rentzos M , Michalopoulou M , Nikolaou C , et al. Serum levels of soluble intercellular adhesion molecule‐1 and soluble endothelial leukocyte adhesion molecule‐1 in Alzheimer's disease. J Geriatr Psychiatry Neurol. 2004;17(4):225‐231. doi:10.1177/0891988704269822 15533994

[13] Kehoe PG , Al Mulhim N , Zetterberg H , Blennow K , Miners JS . Cerebrospinal fluid changes in the renin‐angiotensin system in Alzheimer's disease. J Alzheimers Dis. 2019;72(2):525‐535. doi:10.3233/jad-190721 31594235

[14] Jochemsen HM , Teunissen CE , Ashby EL , et al. The association of angiotensin‐converting enzyme with biomarkers for Alzheimer's disease. Alzheimers Res Ther. 2014;6(3):27. doi:10.1186/alzrt257 24987467 PMC4075229

[15] Montagne A , Barnes SR , Sweeney MD , et al. Blood‐brain barrier breakdown in the aging human hippocampus. Neuron. 2015;85(2):296‐302. doi:10.1016/j.neuron.2014.12.032 25611508 PMC4350773

[16] Guo F , Chen XL , Wang F , Liang X , Sun YX , Wang YJ . Role of angiotensin II type 1 receptor in angiotensin II‐induced cytokine production in macrophages. J Interferon Cytokine Res. 2011;31(4):351‐361. doi:10.1089/jir.2010.0073 21235392

[17] Ruiz‐Ortega M , Ruperez M , Lorenzo O , et al. Angiotensin II regulates the synthesis of proinflammatory cytokines and chemokines in the kidney. Kidney Int Suppl. 2002(82):S12‐22. doi:10.1046/j.1523-1755.62.s82.4.x 12410849

[18] Ballabh P , Braun A , Nedergaard M . The blood‐brain barrier: an overview: structure, regulation, and clinical implications. Neurobiol Dis. 2004;16(1):1‐13. doi:10.1016/j.nbd.2003.12.016 15207256

[19] Brown LS , Foster CG , Courtney JM , NE King , Howells DW , Sutherland BA . Pericytes and neurovascular function in the healthy and diseased brain. Front Cell Neurosci. 2019;13:282. doi:10.3389/fncel.2019.00282 31316352 PMC6611154

[20] Yamazaki Y , Kanekiyo T , Blood‐brain barrier dysfunction and the pathogenesis of Alzheimer's disease. Int J Mol Sci. 2017;18(9). doi:10.3390/ijms18091965 PMC5618614 28902142

[21] Zenaro E , Piacentino G , Constantin G . The blood‐brain barrier in Alzheimer's disease. Neurobiol Dis. 2017;107:41‐56. doi:10.1016/j.nbd.2016.07.007 27425887 PMC5600438

[22] Wang H , Huang L , Wu L , et al. The MMP‐2/TIMP‐2 system in Alzheimer disease. CNS Neurol Disord Drug Targets. 2020;19(6):402‐416. doi:10.2174/1871527319666200812223007 32787764

[23] Hussain AA , Lee Y , Zhang JJ , Francis PT , Marshall J . Disturbed matrix metalloproteinase pathway in both age‐related macular degeneration and Alzheimer's disease. J Neurodegener Dis. 2017;2017:4810232. doi:10.1155/2017/4810232 28197357 PMC5286539

[24] Halliday MR , Rege SV , Ma Q , et al. Accelerated pericyte degeneration and blood‐brain barrier breakdown in apolipoprotein E4 carriers with Alzheimer's disease. J Cereb Blood Flow Metab. 2016;36(1):216‐227. doi:10.1038/jcbfm.2015.44 25757756 PMC4758554

[25] Miners JS , Kehoe PG , Love S , Zetterberg H , Blennow K . CSF evidence of pericyte damage in Alzheimer's disease is associated with markers of blood‐brain barrier dysfunction and disease pathology. Alzheimers Res Ther. 2019;11(1):81. doi:10.1186/s13195-019-0534-8 31521199 PMC6745071

[26] Sagare AP , Sweeney MD , Makshanoff J , Zlokovic BV . Shedding of soluble platelet‐derived growth factor receptor‐β from human brain pericytes. Neurosci Lett. 2015;607:97‐101. doi:10.1016/j.neulet.2015.09.025 26407747 PMC4631673

[27] Nation DA , Sweeney MD , Montagne A , et al. Blood‐brain barrier breakdown is an early biomarker of human cognitive dysfunction. Nat Med. 2019;25(2):270‐276. doi:10.1038/s41591-018-0297-y 30643288 PMC6367058

[28] Miners JS , Schulz I , Love S . Differing associations between Aβ accumulation, hypoperfusion, blood‐brain barrier dysfunction and loss of PDGFRB pericyte marker in the precuneus and parietal white matter in Alzheimer's disease. J Cereb Blood Flow Metab. 2018;38(1):103‐115. doi:10.1177/0271678x17690761 28151041 PMC5757436

[29] Steenland K , Goldstein FC , Levey A , Wharton W . A meta‐analysis of Alzheimer's disease incidence and prevalence comparing African‐Americans and Caucasians. J Alzheimers Dis. 2016;50(1):71‐76. doi:10.3233/jad-150778 26639973 PMC4739787

[30] Howell JC , Watts KD , Parker MW , et al. Race modifies the relationship between cognition and Alzheimer's disease cerebrospinal fluid biomarkers. Alzheimers Res Ther. 2017;9(1):88. doi:10.1186/s13195-017-0315-1 29096697 PMC5668981

[31] Morris JC , Schindler SE , McCue LM , et al. Assessment of racial disparities in biomarkers for Alzheimer disease. JAMA Neurol. 2019;76(3):264‐273. doi:10.1001/jamaneurol.2018.4249 30615028 PMC6439726

[32] Kumar VV , Huang H , Zhao L , et al. Baseline results: the association between cardiovascular risk and preclinical Alzheimer's disease pathology (ASCEND) study. J Alzheimers Dis. 2020;75(1):109‐117. doi:10.3233/JAD-191103 32280088 PMC11380299

[33] McKhann G , Drachman D , Folstein M , Katzman R , Price D , Stadlan EM . Clinical diagnosis of Alzheimer's disease: report of the NINCDS‐ADRDA work group under the auspices of department of health and human services task force on Alzheimer's disease. Neurology. 1984;34(7):939‐944. doi:10.1212/wnl.34.7.939 6610841

[34] Kawas C , Segal J , Stewart WF , Corrada M , Thal LJ . A validation study of the dementia questionnaire. Arch Neurol. 1994;51(9):901‐906. doi:10.1001/archneur.1994.00540210073015 8080390

[35] Leitão MJ , Silva‐Spínola A , Santana I , et al. Clinical validation of the Lumipulse G cerebrospinal fluid assays for routine diagnosis of Alzheimer's disease. Alzheimers Res Ther. 2019;11(1):91. doi:10.1186/s13195-019-0550-8 31759396 PMC6875031

[36] Gobom J , Parnetti L , Rosa‐Neto P , et al. Validation of the LUMIPULSE automated immunoassay for the measurement of core AD biomarkers in cerebrospinal fluid. Clin Chem Lab Med. 2022;60(2):207‐219. doi:10.1515/cclm-2021-0651 34773730

[37] Miners JS , Ashby E , Van Helmond Z , et al. Angiotensin‐converting enzyme (ACE) levels and activity in Alzheimer's disease, and relationship of perivascular ACE‐1 to cerebral amyloid angiopathy. Neuropathol Appl Neurobiol. 2008;34(2):181‐193. doi:10.1111/j.1365-2990.2007.00885.x 17973905

[38] Kehoe PG , Wong S , Al Mulhim N , Palmer LE , Miners JS . Angiotensin‐converting enzyme 2 is reduced in Alzheimer's disease in association with increasing amyloid‐beta and tau pathology. Alzheimers Res Ther. 2016;8(1):50. doi:10.1186/s13195-016-0217-7 27884212 PMC5123239

[39] Lehmann S , Dumurgier J , Ayrignac X , et al. Cerebrospinal fluid A beta 1‐40 peptides increase in Alzheimer's disease and are highly correlated with phospho‐tau in control individuals. Alzheimers Res Ther. 2020;12(1):123. doi:10.1186/s13195-020-00696-1 33008460 PMC7532565

[40] Tijms BM , Vermunt L , Zwan MD , et al. Pre‐amyloid stage of Alzheimer's disease in cognitively normal individuals. Ann Clin Transl Neurol. 2018;5(9):1037‐1047. doi:10.1002/acn3.615 30250861 PMC6144448

[41] Welge V , Fiege O , Lewczuk P , et al. Combined CSF tau, p‐tau181 and amyloid‐beta 38/40/42 for diagnosing Alzheimer's disease. J Neural Transm (Vienna). 2009;116(2):203‐212. doi:10.1007/s00702-008-0177-6 19142572

[42] Sundelöf J , Sundström J , Hansson O , et al. Higher cathepsin B levels in plasma in Alzheimer's disease compared to healthy controls. J Alzheimers Dis. 2010;22(4):1223‐1230. doi:10.3233/jad-2010-101023 20930303

[43] Olsson B , Lautner R , Andreasson U , et al. CSF and blood biomarkers for the diagnosis of Alzheimer's disease: a systematic review and meta‐analysis. Lancet Neurol. 2016;15(7):673‐684. doi:10.1016/s1474-4422(16)00070-3 27068280

[44] Bibl M , Mollenhauer B , Lewczuk P , et al. Cerebrospinal fluid tau, p‐tau 181 and amyloid‐β38/40/42 in frontotemporal dementias and primary progressive aphasias. Dement Geriatr Cogn Disord. 2011;31(1):37‐44. doi:10.1159/000322370 21135556

[45] Gabelle A , Roche S , Gény C , et al. Decreased sAβPPβ, Aβ38, and Aβ40 cerebrospinal fluid levels in frontotemporal dementia. J Alzheimers Dis. 2011;26(3):553‐563. doi:10.3233/jad-2011-110515 21709372

[46] Janelidze S , Zetterberg H , Mattsson N , et al. CSF Aβ42/Aβ40 and Aβ42/Aβ38 ratios: better diagnostic markers of Alzheimer disease. Ann Clin Transl Neurol. 2016;3(3):154‐165. doi:10.1002/acn3.274 27042676 PMC4774260

[47] Young AL , Oxtoby NP , Daga P , et al. A data‐driven model of biomarker changes in sporadic Alzheimer's disease. Brain. 2014;137(Pt 9):2564‐2577. doi:10.1093/brain/awu176 25012224 PMC4132648

[48] Xiong C , Jasielec MS , Weng H , et al. Longitudinal relationships among biomarkers for Alzheimer disease in the adult children study. Neurology. 2016;86(16):1499‐1506. doi:10.1212/wnl.0000000000002593 27009258 PMC4836885

[49] Garrett SL , McDaniel D , Obideen M , et al. Racial disparity in cerebrospinal fluid amyloid and tau biomarkers and associated cutoffs for mild cognitive impairment. JAMA Netw Open. 2019;2(12):e1917363. doi:10.1001/jamanetworkopen.2019.17363 31834392 PMC6991300

[50] Shi Y , Yamada K , Liddelow SA , et al. ApoE4 markedly exacerbates tau‐mediated neurodegeneration in a mouse model of tauopathy. Nature. 2017;549(7673):523‐527. doi:10.1038/nature24016 28959956 PMC5641217

[51] Barnes LL . Biomarkers for Alzheimer dementia in diverse racial and ethnic minorities—a public health priority. JAMA Neurology. 2019;76(3):251‐253. doi:10.1001/jamaneurol.2018.3444 30615027

[52] Ahmad S , Ashktorab H , Brim H , Housseau F . Inflammation, microbiome and colorectal cancer disparity in African‐Americans: are there bugs in the genetics. World J Gastroenterol. 2022;28(25):2782‐2801. doi:10.3748/wjg.v28.i25.2782 35978869 PMC9280725

[53] Goldstein FC , Zhao L , Steenland K , Levey AI . Inflammation and cognitive functioning in African Americans and Caucasians. Int J Geriatr Psychiatry. 2015;30(9):934‐941. doi:10.1002/gps.4238 25503371 PMC4682666

[54] Hyatt TC , Phadke RP , Hunter GR , Bush NC , Muñoz AJ , Gower BA . Insulin sensitivity in African‐American and white women: association with inflammation. Obesity. 2009;17(2):276‐282. doi:10.1038/oby.2008.549 19039315 PMC2748773

[55] Kiely M , Lord B . Ambs S . Immune response and inflammation in cancer health disparities. Trends Cancer. 2022;8(4):316‐327. doi:10.1016/j.trecan.2021.11.010 34965905 PMC8930618

[56] Simons RL , Lei MK , Klopack E , Zhang Y , Gibbons FX , Beach SRH . Racial discrimination, inflammation, and chronic illness among African American women at midlife: support for the weathering perspective. J Racial Ethn Health Disparities. 2021;8(2):339‐349. doi:10.1007/s40615-020-00786-8 32488825 PMC8183614

[57] Villablanca AC , Warford C , Wheeler K . Inflammation and cardiometabolic risk in African American women is reduced by a pilot community‐based educational intervention. J Womens Health. 2016;25(2):188‐199. doi:10.1089/jwh.2014.5109 26263081

[58] Gillespie SL , Christian LM , Mackos AR , et al. Lifetime stressor exposure, systemic inflammation during pregnancy, and preterm birth among Black American women. Brain Behav Immun. 2022;101:266‐274. doi:10.1016/j.bbi.2022.01.008 35031400 PMC8885874

[59] Sistrunk C , Tolbert N , Sanchez‐Pino MD , et al. Impact of federal, state, and local housing policies on disparities in cardiovascular disease in Black/African American men and women: from policy to pathways to Biology. Front Cardiovasc Med. 2022;9:756734. doi:10.3389/fcvm.2022.756734 35509276 PMC9058117

[60] Boots EA , Feinstein DL , Leurgans S , et al. Acute versus chronic inflammatory markers and cognition in older black adults: results from the minority aging research study. Brain Behav Immun. 2022;103:163‐170. doi:10.1016/j.bbi.2022.04.014 35439553 PMC9704497

[61] Jia L , Xu H , Chen S , et al. The APOE ε4 exerts differential effects on familial and other subtypes of Alzheimer's disease. Alzheimers Dement. 2020;16(12):1613‐1623. doi:10.1002/alz.12153 32881347 PMC7984370

[62] Asby D , Boche D , Allan S , Love S , Miners JS . Systemic infection exacerbates cerebrovascular dysfunction in Alzheimer's disease. Brain. 2021;144(6):1869‐1883. doi:10.1093/brain/awab094 33723589 PMC8320299

[63] Trombetta BA , Kivisäkk P , Carlyle BC , et al. Plasma biomarkers of neuroinflammation and vascular injury predict cognitive decline in patients with mild cognitive impairment. Alzheimers Dement. 2020;16(S5):e046134. doi:10.1002/alz.046134

[64] Jiang H , Hampel H , Prvulovic D , et al. Elevated CSF levels of TACE activity and soluble TNF receptors in subjects with mild cognitive impairment and patients with Alzheimer's disease. Mol Neurodegener. 2011;6:69. doi:10.1186/1750-1326-6-69 21978728 PMC3206445

[65] Zhao A , Li Y , Deng Y . TNF receptors are associated with tau pathology and conversion to Alzheimer's dementia in subjects with mild cognitive impairment. Neurosci Lett. 2020;738:135392. doi:10.1016/j.neulet.2020.135392 32947003

